# Effect of iPS cell culture medium on the differentiation potential of induced cardiac tissues

**DOI:** 10.1038/s41598-025-13259-x

**Published:** 2025-08-03

**Authors:** Yoshiki Nakashima, Masayoshi Tsukahara

**Affiliations:** Research and Development Center, CiRA Foundation, Nakanoshima Qross, Osaka, 530-005 Japan

**Keywords:** Induced pluripotent stem cells (iPSCs), Cardiac tissues, Atrial natriuretic peptides (ANP), Brain natriuretic peptide (BNP), Regenerative medicine, Stem-cell biotechnology, Tissue engineering, Pluripotent stem cells, Stem-cell differentiation

## Abstract

The present study focused on the culture medium of induced pluripotent stem cells (iPSCs) prior to the use of cardiomyocytes differentiation induction medium (pre-culture medium). Seven types (Nos. 1-7) of StemFit AK03 medium (Ajinomoto) for clinical iPSCs with varying compositions were prepared as pre-culture medium. The cardiac muscle troponin T (cTnT) positivity of No. 1 (StemFit AK03 medium) was 84%, No. 3 (similar to E8 medium) was 89%, No. 2 (similar to E8 medium) was 91%, No. 5 (similar to EB Formation medium) was 95%, when using differentiation induction medium prepared with known components available for clinical cell production. The formation of cardiac tissues was assessed by evaluating the expression levels of specific markers, including cTnT, atrial natriuretic peptides (ANP), and pro-B-type natriuretic peptide (proBNP). The results demonstrated that cardiac tissue with high protein expression levels of cTnT and ANP was formed when similar to E8 medium as pre-culture medium.

**Supplementary Information:**

The online version contains supplementary material available at 10.1038/s41598-025-13259-x.

## Introduction

The induced pluripotent stem cell (iPSC)^1,2^ culture medium^3–5^ has been optimized for continuous maintenance of iPSC passages^6^. Consequently, high levels of pluripotency may inadvertently interfere with the process of differentiation induction immediately preceding this process. Undifferentiated iPSCs (induced pluripotent stem cells) undergo differentiation medium to either ectoderm, mesoderm, or endoderm during the initial 2 days of the differentiation induction protocol before proceeding to the differentiation maturation process. During the initial phase of differentiation induction, iPSCs must concurrently adjust to the composition of the medium and the differentiation induction process. Consequently, iPSCs experience a period of significant culture adaptation stress^7^. The hypothesis was that the differentiation of iPSCs would be further promoted, and the final differentiation induction efficiency would be improved if the culture adaptation stress of iPSCs could be alleviated. In this study, we sought to evaluate the impact of pre-culture medium on the process of cardiomyocyte differentiation induction from iPSCs.

In individual development, during the initial phases of embryogenesis, the induction of differentiation in the ectoderm, mesoderm, and endoderm is a concurrent process, contingent upon the specific location of the germ. The embryoid body (EB) formation method^8,9^ is a technique that replicates the developmental process of an organism in a laboratory setting. It involves the cultivation of EBs from a spherical cluster of pluripotent stem cells, thereby simulating the in vivo process of ontogeny. Tissues that have undergone differentiation from EBs are referred to as organoids^10–12^in which cell-cell interactions play a pivotal role in the processes of differentiation and maturation. FGF2-free medium is used for EB formation^13^. Therefore, the optimal pre-culture medium may depend on whether the pluripotent cells are iPSCs or EBs composed of iPSCs. Our focus shifted to analysis cardiac development as an assessment of the pre-culture medium.

In cardiac development, ISL1 + multipotent embryonic progenitor cells^14,15^ have been identified as cells capable of differentiating into all major cardiac cell types and at least three cardiac cell types: cardiomyocytes, smooth muscle and endothelium^16–19^. It is known that during heart development, cells derived from the proepicardium, a mesodermal projection that arises near the liver, later give rise to the coronary arteries and the epicardium that covers the surface of the heart^20^. It has been shown that the proper cardiac morphogenesis is contingent upon the contribution of proepicardium (PE) cells that form in the vicinity of the liver primordium and that co-culture of epicardial cells and cardiomyocytes further promotes the maturation of cardiac tissue^18,21^. Therefore, cardiac differentiation techniques employing iPSCs can enhance efficiency through the approximation of heart formation during embryogenesis.

On the other hand, when cardiomyocytes are induced from iPSCs in general, it is difficult to induce the differentiation of only a single type of cardiomyocyte with current differentiation induction protocols^22^. At least two cell types, clear adherent cells and raised brown cells, are induced to differentiate and observed in the cell culture dish. This study demonstrated that these cells have cell-cell interactions that promote cardiac tissue maturation. In the current protocol to induce cardiomyocyte differentiation from iPSCs, the expression levels of the cardiomyocyte marker troponin T, the ventricular marker MLC2v and the atrial marker MLC2a are the main targets of evaluation^23,24^. The evaluation of site-specific markers of these organs is an important indicator in assessing the location of organs in cardiac embryology^25^.

On the other hand, since hormones have bioactive functions, they could enhance the cardiac organ formation from iPSCs, and in particular hormones secreted by cardiac organs. The hormones secreted by the heart are atrial natriuretic peptide (ANP), mainly secreted by the atria, and brain natriuretic peptide (BNP), mainly synthesized by the ventricles. ANP was discovered in 1984 and BNP in 1988 by Matsuo and Kangawa et al.^26,27^. Hormones identified in the search for endogenous ligands for orphan GPCRs, such as ANP and BNP, have been studied in detail by a team from the National Cerebral and Cardiovascular Center in Japan^28,29^ and Dr. Olivier Civelli’s team^30^.

In this study, in addition to the existing evaluation of the efficiency of iPSC-derived cardiomyocytes (iCMs) differentiation induction using the expression levels of the cardiomyocyte marker troponin T, the ventricular marker MLC2v and the atrial marker MLC2a as indicators, ANP and BNP were added to assess cardiac tissues formation derived from iPSCs.

Next, the method of inducing differentiation into cardiac tissue was evaluated, focusing on pre-culture medium. In the early stages of pioneering differentiation induction protocols, the EB method using a medium containing 20% FBS produced differentiated cell types from all three germ layers, including cardiomyocytes. This contributed to the development of protocols for inducing cardiomyocyte differentiation, although the method was inefficient^31–36^. In one hand, EBs are spheroids that exhibit pluripotency, and high nutrient media with a high content of growth factors and albumin, such as fetal bovine serum (FBS), are used as the culture medium. Recent protocols allow iPSCs to form a pluripotent spheroid when cultured in a pluripotency maintenance medium, such as Essential 8 medium, on a low-adherent plate. The spheroid is then incubated in EB-forming medium containing 20% KnockOut Serum Replacement (KOSR) (77% DMEM + 20% KOSR + 1% NEAA + 0.1% β-mercaptoethanol + 1% GlutaMAX + 1% Pen/Strep) to produce EBs^37^. KOSR includes growth factors such as bFGF (basic fibroblast growth factor) and EGF (epidermal growth factor), the buffer HEPES, and proteins such as albumin, transferrin, and amino acids. It also contains vitamins and trace elements such as zinc and iron. The Essential 8 medium consists of DMEM/F12, NaHCO₃, L-ascorbic acid, selenium, transferrin, TGF-β1, and FGF2. The main difference between the Essential 8 medium and the EB formation medium is that the former does not contain fetal bovine serum (FBS) substitutes, such as KOSR.

On the other hand, low nutrient media, such as Essential 8 medium without albumin, are used in the composition of media that can selectively culture iPSCs. Both EBs and iPSCs are pluripotent cells, but the composition of the optimal culture medium is very different. In this study, a pre-culture medium was prepared that approximated the composition of the medium for EB formation and the Essential 8 medium. The differentiation potential of cultured iPSCs was evaluated in the process of inducing cardiomyocyte differentiation using different pre-culture media.

## Materials and methods

### Materials and resources used

StemFit AK03N was purchased from Ajinomoto Healthy Supply Co., Inc. (Tokyo, Japan). iMatrix-511 was purchased from Matrixome, Inc. (Osaka, Japan). A 10-mmol/L Y-27632 solution, D-PBS (-), and 0.5 mol/ L EDTA solution (pH 8.0) were purchased from Nacalai Tesque (Kyoto, Japan). Essential 8 medium, RPMI 1640 medium, B-27 supplement, minus insulin, B-27 supplement (50 ×), TrypLE Select Enzyme (1 ×) were purchased from Thermo Fisher Scientific K.K. (Kanagawa, Japan). mTeSR Plus-cGMP medium and Clone R were purchased from STEMCELL Technologies (Vancouver, Canada). Biolaminin 521 LN (LN521) was purchased from BioLamina (Sundbyberg, Sweden). XAV939, a Wnt pathway inhibitor, was purchased from FUJIFILM Wako Pure Chemical Corporation (Osaka, Japan). Glycogen synthase kinase-3 inhibitor CHIR 99021 was purchased from Axon Medchem LLC (Reston, VA). Alexa Fluor 647 mouse anti-cardiac troponin T (BD565744), Alexa Fluor 647 mouse IgG1, κ isotype control (BD566011) were purchased from Becton, Dickinson and Company (Franklin Lakes, NJ, USA). MYL2 antibody (7C9) (sc-517244), ANP antibody (F-2) (sc-515701) were purchased from Santa Cruz Biotechnology, Inc. (Dallas, TX, USA). NT-proBNP antibody (5B6cc), BNP antibody (50E1cc), and MYL7 antibody (OTI4E7) were purchased from Bio-Techne (Minneapolis, MN, USA). The Cellstain-Hoechst 33342 solution was purchased from Dojindo Laboratories (Kumamoto, Japan). SuperQuality Petri dishes and aspiration pipettes were purchased from Sumitomo Bakelite Co. Ltd. (Tokyo, Japan).

### Maintenance culture of hiPSCs

The standard protocol for culturing iPSCs is the method developed by Dr. Nakagawa and Prof. Yamanaka’s group at CiRA^38^. These experimental techniques have been previously reported by us^39,40^. The hiPSC lines CFiS-S01 were established by Shinya Yamanaka (CiRA Foundation) and obtained from CiRA Foundation (Kyoto, Japan).

#### Culture method using iMatrix-511 + StemFitAK03 medium

The iPSCs were cultured according to a publicly available method (CiRA_Ff-iPSC_protocol_Eng_v140310) (https://www.cira.kyoto-u.ac.jp/j/research/img/protocol/Ff-iPSC-culture_protocol_E_v140311.pdf)^38,41^. Coating with iMatrix-511 (175 µg/0.35 mL/tube) was performed by adding 9.6 µL to each well of a 6-well plate. The iMatrix-511 (9.6 µL) was first diluted in 1.5 mL of PBS and then added as a coating solution to each well of a 6-well plate. A minimum coating time of at least 1 h at 37 °C was used.

#### Culture method using LN521 + mTeSR Plus-cGMP medium

hiPSCs that had been cultured on laminin-coated (LN521) were added to six-well plates (1.3 × 10^4^ cells/well); mTeSR Plus-cGMP medium containing 150 µL of CloneR2 was added to each well to reach a final volume of 1.5 mL in each well. Cells were incubated at 37 °C in 5% CO_2_, according to the manufacturer’s instructions (https://cdn.stemcell.com/media/files/manual/10000007757-Maintenance_of_Human_Pluripotent_Stem_Cells_in_mTeSR_Plus.pdf). When Essential 8 medium was used as the pre-culture medium, it was replaced with Essential 8 medium only on the last day of culture.

### Induction of myocardial differentiation using commercially available differentiation induction kits

hiPSCs cultured on laminin-coated (iMatrix-511) were added to six-well plates at a density of 1.3 × 10^4^ cells per well. StemFit AK03N supplemented with 10 µM Y27632 was added to to each well, bringing the final volume of 1.5 mL. The cells were then incubated at 37 °C in 5% CO_2_. On days -6, -4, and -2 prior to differentiation, the medium was replaced. It was replaced with 1.5 mL of room temperature StemFit AK03N per well. To induce cardiomyocyte differentiation into myocardial cells, hiPSCs were grown to confluence in six-well plates in StemFit AK03N medium on a support using a PSC Cardiomyocyte Differentiation Kit, according to the manufacturer’s instructions (https://assets.thermofisher.com/TFS-Assets/LSG/manuals/MAN0014509_psc_cardiomyocyte_diff_PI.pdf) (Thermo Fisher Scientific K.K.)^31,42,43^ or STEMdiff Cardiomyocyte Differentiation and Maintenance Kits, according to the manufacturer’s instructions (https://cdn.stemcell.com/media/files/pis/DX21496-PIS_1_0_0.pdf?_ga=2.262974152.598384201.1535506696-776122060.1533191873) (STEMCELL Technologies Inc.). Specifically, the cells were cultured with solution A for two days, followed by solution B for another two days. Then, the cells myocardial differentiation potential was assessed using cells that had been cultured in solution C for 5–7 days. The cultures were performed in an incubator at 37 °C with 20% O_2_ and 5% CO_2_.

### Trilineage differentiation

These experimental techniques have been previously reported by us^40^. The STEMdiff Trilineage Differentiation Kit (ST-05230; (Stemcell Technologies), was used. The cells were cultured for two days using STEMdiff Trilineage Ectoderm Medium to induce differentiation of iPSCs into ectoderm. The differentiation of the iPSCs into the mesoderm lineage was induced by culturing the cells for two days using the STEMdiff Trilineage Mesoderm Medium. The differentiation of the iPSCs into endoderm was induced by culturing the cells for two days using the STEMdiff Trilineage Endoderm Medium.

### Induction of myocardial differentiation using custom media

The method published in 2013 by Lian et al. was used as a standard protocol for the induction of cardiomyocyte differentiation^44^. These experimental techniques have been previously reported by us^45^. (1) hiPSCs cultured on laminin-coated (iMatrix-511) were added to six-well plates at a density of 1.3 × 10^4^ cells/well. StemFit AK03N supplemented with 10 µM Y27632 was added to each well, bringing the final volume to 1.5 mL. The cells were then incubated at 37 °C in 5% CO_2_. (2) Prior to differentiation, on days -6, -4, and -2, the medium was replaced with 1.5 mL of room temperature StemFit AK03N per well. (3) On day 0 of differentiation, 12 µM CHIR99021 was added to each well in 4 mL of RPMI/B-27 without insulin. (4) After 24 h (day 1 of differentiation), the medium in each well was replaced with 4 mL of room temperature RPMI/B-27 without insulin. The plates were returned to the incubator. (5) On day 3 of differentiation, 2 µM XAV939 was added to each well. (6) On day 5, the medium was removed from each well, and 4 mL of room temperature RPMI/B-27 without insulin was added to each well. The plate was then returned to the incubator. (7) On day 7 of differentiation and every 3 days thereafter, replace the medium in each well with 4 mL of fresh RPMI/B-27, and then incubate.

### Pre-culture medium for clinical iPSCs

StemFit AK03 (Ajinomoto) was selected as a material that could be used to produce cells for clinical use, and the preculture medium was prepared by varying the composition of its solution A (400 mL), B (100 mL), and C (2 mL). The preculture medium was prepared by mixing the medium with the following amounts of liquid.

StemFit AK03: No. 1 (liquid A 400 mL + liquid B 100 mL + liquid C 2 mL), No. 2 (400 mL solution A + 100 mL solution B + 0.2 mL solution C), No. 3 (400 mL solution A + 25 mL solution B + 0.5 mL solution C), No. 4 (liquid A 40 mL + liquid B 10 mL + liquid C 2 mL), No. 5 (100 mL liquid A + 100 mL liquid B + 2 mL liquid C), No. 6 (liquid A 80 mL + liquid B 20 mL + liquid C 2 mL), No. 7 (liquid A 200 mL + liquid B 50 mL + liquid C 2 mL).

### PluriTest

We used the PluriTest analysis service from Life Technologies Corporation (Carlsbad, CA, USA)^46^. This assay assesses 36,000 transcripts and variants against a reference set of > 450 samples for gene expression analysis.

The transcriptomes of all samples were analyzed and processed in the PluriTest algorithm to generate pluripotency and novelty scores. The pluripotency score is based on many samples (pluripotent, somatic, and tissue) in the stem cell model matrix, which consists of an extensive reference set of > 450 cell/tissue types, including 223 hESC lines (Stem Cell Matrix-2’ (SCM2) database)^47^ lines, 41 iPSC lines, somatic cells, and tissues.

RNA purification with this system involves cell preparation using a PureLink RNA Mini Kit (Catalog #12183025;(Thermo Fisher Scientific, Waltham, MA, USA) and quantification using the NanoDrop (Thermo Fisher Scientific, Waltham, MA, USA). The GeneChip for the PluriTest is prepared using 100 ng of total RNA.

### KaryoStat

We used the KaryoStat assay service (https://assets.thermofisher.com/TFS-Assets/BID/posters/karyostat-service-alternative-to-karyotyping-stemcells-poster.pdf) provided by Life Technologies Corporation (Carlsbad, CA, USA)^46^. The KaryoStat assay allows for digital visualization of chromosomal aberrations with a resolution similar to g-banding karyotyping. The size of the structural aberration that can be detected is > 2 Mb for chromosomal gains and > 1 Mb for chromosomal losses. The KaryoStat array is optimized for balanced whole-genome coverage with a low-resolution DNA copy number analysis, and the assay covers all 36,000 Ref Seq genes, including 14,000 OMIM targets. The assay enables the detection of aneuploidies, submicroscopic aberrations, and mosaic events.

Genomic DNA (gDNA) was prepared using the Genomic DNA Purification Kit (Catalog K 0512;(Qiagen, Hilden, Germany) and quantified using the Qubit dsDNA BR Assay Kit (Catalog Q 32850GeneChip Preparation; (Thermo Fisher Scientific, Waltham, MA, USA). A total of 250 ng of gDNA was used to prepare the GeneChip for KaryoStat according to the instructions, which is an array that looks for copy number variants and single nucleotide polymorphisms across the genome.

gDNA was processed according to the manufacturer’s protocol. In brief, 250 ng of gDNA was digested with the NspI restriction enzyme. Then, the digested DNA was ligated to the NspI adapter and amplified by PCR. The PCR products were then purified and fragmented using DNase I, after which they were end-labeled with biotin and hybridized overnight to KaryoStat arrays (Thermo Fisher Scientific) in a GeneChip Hybridization Oven 645 (Thermo Fisher Scientific). The arrays were then washed and stained using a GeneChip Fluidics Station 450 (Thermo Fisher Scientific) and scanned using a GeneChip Scanner 3000 7G (Thermo Fisher Scientific). The GeneChip Command Console software program generated scanned data files, which were then analyzed using Chromosome Analysis Suite v4.3 (ChAS). The analysis considered 1–2 MB for gains/losses and 5 MB for LOH(loss of heterozygosity)/AOH(absence of heterozygosity).

### ECLIA (electro chemiluminescence immunoassay)

The culture supernatant was concentrated 10-fold with Amicon-Ultra15 (10 K) (Merck Millipore). The e801, a module of 8000 series^48^ (Roche Diagnostics, Mannheim, Germany) was used as the measuring instrument. Analysis was performed by SRL, Inc. (Tokyo, Japan).

### Flow cytometry

Cell flow cytometry was performed using a Spectral Cell Analyzer SA3800 (Sony Corporation, Tokyo, Japan) according to the manufacturer’s instructions.

Method for staining cell membrane antigens: Briefly, iPSCs (1 × 10^6^) were added to 1.0 mL of BD Cytofix Fixation Buffer (BD Biosciences, Franklin Lakes, New Jersey). Fixed cells were placed in microtubes and allowed to stand at room temperature for 20 min, then centrifuged at 300 g for 5 min to remove the supernatant. After centrifugation, PBS (2% FBS) containing 0.1 mL of antibody (1/100 dilution) was added to the cell pellets of iPSCs (1 × 10^6^) and mixed well. Primary antibodies were as follows: Alexa Fluor 647 mouse anti-cardiac troponin T (BD Biosciences; 565744), Alexa Fluor 647 mouse IgG1, κ isotype control (BD Biosciences; 566011), MYL2 antibody (7C9) (Santa Cruz Biotechnology; sc-517244), ANP antibody (F-2) (Santa Cruz Biotechnology; sc-515701), NT-proBNP antibody (Bio-Techne; 5B6cc), BNP antibody (Bio-Techne; 50E1cc), MYL7 antibody (Bio-Techne; OTI4E7). The sample was incubated with the antibody for 30 min at room temperature. During the antibody reaction, the cells are placed in microtubes and allowed to stand at room temperature for 30 min, then centrifuged at 300 g for 5 min to remove the supernatant. After centrifugation, 1 mL of PBS (2% FBS) was added to the cell pellet, mixed well, and centrifuged again at 300 g for 5 min. This antibody washing procedure was repeated twice. After centrifugation, 300 µL of PBS (2% FBS) was added to the cell pellet and mixed with the cell suspension, which was then used for flow cytometry.

### Immunofluorescence staining analyses

These experimental techniques have been previously reported by us^49^. Cells were washed with PBS, immersed in Cytofix fixation buffer (BD Biosciences), and fixed for 10 min. Liquid 2% FBS added to PBS was used as an antibody diluent. Immunofluorescence staining was performed using the following specific antibodies: Alexa Fluor 647 mouse anti-cardiac troponin T (BD Biosciences; 565744), ANP antibody (F-2) (Santa Cruz Biotechnology; sc-515701), and NT-proBNP antibody (Bio-Techne; 5B6cc). Cellstain-Hoechst 33342 solution (DOJINDO LABORATORIES, Kumamoto, Japan) was used for the nuclear staining of the cells. Images were captured with a fluorescence microscope (BZ-X800; Keyence, Osaka, Japan).

### Real-time polymerase chain reaction (PCR)

These experimental techniques have been previously reported by us^39,40^. RNA was prepared for quantitative PCR using a SuperPREP II Cell Lysis & RT Kit (TOYOBO CO., LTD., Osaka, Japan) according to the manufacturer’s instructions. Real-time PCR was performed using a StepOnePlus system (Life Technologies, Carlsbad, CA, USA). Luna Universal qPCR Master Mix (New England Biolabs, Inc., Ipswich, MA, USA) was used according to the manufacturer’s instructions. The PCR protocol was as follows: (1) initial denaturation at 95 °C for 10 min, (2) denaturation at 95 °C for 15 s, (3) primer annealing at 60 °C for 60 s. Steps (2)-(3) were repeated 40 times. Then, (4) denaturation at 95 °C for 15 s, primer annealing at 60 °C for 60 s, and denaturation at 95 °C for 15 s. A TaqMan Array 96-well FAST plate (Human Stem Cell Pluripotency and Human Factors Promoting Card, Applied Biosystems) was used for mRNA expression analysis. TaqMan Fast Advanced Master Mix (Thermo Fisher Scientific) was used according to the manufacturer’s instructions. The PCR protocol was as follows: (1) Denature at 95 °C for 20 s. (2) Anneal primers at 60 °C for 20 s. Repeat steps (1)-(2) 40 times.

For the RT-PCR data analysis using a TaqMan Array 96-Well FAST Plate, the housekeeping genes *18S*, *GAPDH*, *HPRT1*, and *GUSB* were used. The maximum CT value was set to 40. The ΔCT value for targets that were not detected was calculated by subtracting the average CT value of the 4 housekeeping genes from the maximum CT value (40). The ΔCT value of each target gene was calculated by subtracting the average CT value of the four housekeeping genes from its CT value under each culture condition. The ΔΔCT values were calculated by subtracting the average ΔCT values of the different genes under control culture conditions from the ΔCT values of the different genes under each culture condition. The ΔΔCT values were calculated using an Excel software program (Microsoft Corporation, Redmond, WA, USA).

Expression was calculated using the ΔΔCt method. Target gene expression was normalized to housekeeping gene expression. Primers were designed to optimize the sequence for each of the following targets: human *β-actin*, *Brachyury* (*T*), *KDR*,* NKX2*.5, *ISL1*,* and Troponin T* (*cTnT*). Gene names were obtained from the U.S. National Library of Medicine (NIH) website (https://www.ncbi.nlm.nih.gov/pubmed/). Primers for human *β-actin*, *Brachyury* (*T*), *KDR*,* NKX2*.5, *ISL1*, and *Troponin T* (*cTnT*) were designed using the Primer 3 Plus application (http://www.bioinformatics.nl/cgi-bin/primer3plus/primer3plus.cgi). The primers used for PCR were previously described^50^.

### DNA methylation analysis

The analysis was performed by UNUTECH (Chiba, Japan). Lysis buffer (containing Proteinase K) was added to the frozen cell pellets and incubated at 55 °C until the cells were completely lysed. TE-saturated phenol (Nippon Gene Co., Ltd.) and phenol/chloroform/isoamyl alcohol (25:24:1) (Nippon Gene Co., Ltd.) were used to extract cell-derived genomic DNA. The extracted cell-derived genomic DNA was dissolved in TE buffer. Bisulfite treatment of cell-derived genomic DNA was performed using the EpiTect Bisulfite Kit (QIAGEN). Bisulfite treated cell-derived genomic DNA was used as a template, and PCR products were prepared using PCR primers for methylation analysis. PCR products were purified using the FavorPrep GEL/PCR Purification Mini Kit (FAVORGEN). The purified PCR products were subjected to 100 V electrophoresis in TAE buffer on a 2% agarose gel. The purified PCR products were cloned into pBluescript II SK(+) (Stratagene) using NEBuilder HiFi DNA Assembly Master Mix (New England biolabs). The prepared plasmid solution was introduced into competent cells via transformation (E. coli JM109) by heat treatment (42 °C for 30 s) and plated onto ampicillin-containing LB agar plates (UNUTECH, Chiba, Japan). After overnight incubation at 37 °C, E. coli colonies were picked up and incubated in 100 µL ampicillin-containing LB medium (UNUTECH, Chiba, Japan). Colony PCR was performed using PrimeSTAR GXL DNA Polymerase (Takara Bio Inc.) in a 15 µL reaction system with 2 µL of E. coli culture as template. PCR reactions were performed on the GeneAmp PCR System 9700 (Applied Biosystems). Electrophoresis of the resulting PCR products was performed at 100 V in TAE buffer using on a 2% agarose gel to confirm the band size of each E. coli clone. E. coli clones carrying the plasmid were expanded in 1 mL of ampicillin-containing LB medium (UNUTECH, Chiba, Japan) from E. coli culture medium stored at 4 °C. Plasmids were extracted from the expanded E. coli cultures by the alkaline SDS method. The purified plasmids were used as templates for sequencing reactions using the QuantumDye Terminator v3.1 Cycle Sequencing Kit (TOMY Digital Biology, Inc.). Sequencing reactions were performed under the following conditions: denaturation at 96 °C for 10 s, annealing at 50 °C for 5 s, and extension at 60 °C for 2 min. 25 cycles of sequencing were performed on an ABI PRISM 3130xl Genetic Analyzer (Applied Biosystems). The sequence was compared with the sequence after bisulfite treatment to determine the presence or absence of methylation of CpG sites in the genomic DNA sequence.

### Statistical analyses

Statistical analyses were performed using Student’s t-test to compare the means of two samples. Analyses of multiple groups (i.e., more than two groups) were performed using one-way and two-way analyses of variance with the StatPlus software program (AnalystSoft, Walnut, CA, USA). Statistical significance was set at *P < 0.05 or **P < 0.01 for all tests. Data shown are representative examples of two independent experiments.

## Results

### Differentiation marker mRNA expression affected by different iPSC culture media

**Fig. 1:**
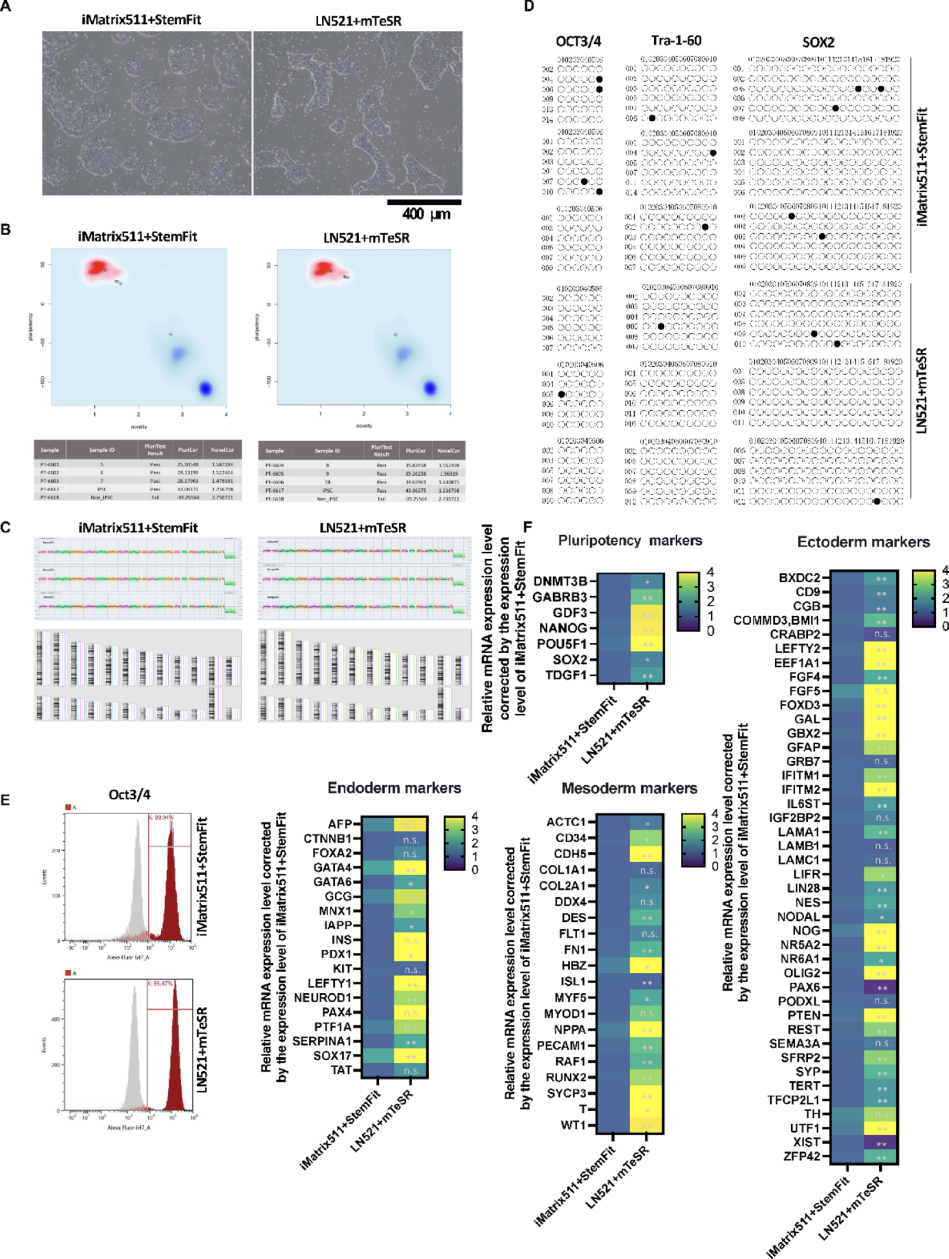
The quality of iPSCs was compared using two types of culture media. (**A**) Two different methods, StemFit AK03 (Ajinomoto) + iMatrix-511 (Matrixome)[iMatrix511 + StemFit] and mTeSR Plus-cGMP (STEMCELL Technologies) + Biolaminin 521 LN (BioLamina)[LN521 + mTeSR], were used to culture the iPSCs. Differences in iPSC colony morphology were observed between the two culture methods. (**B**) PluriTest scores were 27.4 ± 1.1 for iMatrix511 + StemFit and 35.2 ± 0.5 for LN521 + mTeSR. *n* = 3. LN521 + mTeSR was significantly higher than iMatrix511 + StemFit. (**C**) KaryoStat assays revealed no genetic or karyotypic mutations in both iMatrix511 + StemFit and LN521 + mTeSR. *n* = 3. (**D**) Bisulfite sequence analysis of genomic DNA. Bisulfite sequencing analysis of the OCT3/4, Tra-1-60, and SOX2 promoter regions in iPSCs cultured in iMatrix511 + StemFit or LN521 + mTeSR. Open circles indicate unmethylated and closed circles indicate methylated CpG dinucleotides. *n* = 3. (**E**) Results of flow cytometry analysis of the OCT3/4 positive rate of iPSCs. The OCT3/4 positive rate of iPSCs cultured with iMatrix511 + StemFit was 88.9%, and that of iPSCs cultured with LN521 + mTeSR was 95.4%. The corresponding isotype is shown in gray. (**F**) mRNA expression analysis using TaqMan Array Human Stem Cell Pluripotency. iPSCs cultured under LN521 + mTeSR conditions expressed higher mRNA levels of endoderm marker SOX17 and mesoderm marker Brachyury (T) than iMatrix511 + StemFit conditions. *n* = 6.

In this experiment, the iPSCs were PBMC-derived iPSCs (CFiS-S01 strain), which were generated using the same protocol as clinical iPSCs. The quality of iPSCs was compared using two different culture media: StemFit AK03 (Ajinomoto) + iMatrix-511 (Matrixome) and mTeSR Plus-cGMP (STEMCELL Technologies) + Biolaminin 521 LN (BioLamina). The iPSC colony morphology of iMatrix511 + StemFit cells was an ES cell-like colony, while that of LN521 + mTeSR cells was a peninsula-like colony^49^ (Fig. 1A). Our previous reports have shown that peninsula-like colonies have reduced cell polarity compared to ES cell colonies. They are better adapted to the 3D environment and maintain pluripotency. The mRNA expression levels of iPSCs cultured under two different culture conditions were determined to be pluripotent stem cells based on the results of the PluriTest^47^ (Fig. 1B). KaryoStat analysis revealed no karyotypic or genetic alterations in the two culture conditions (Fig. 1C). DNA methylation analysis of the promoter regions of the undifferentiated markers OCT3/4, Tra-1-60 and SOX2 showed that all promoter regions were demethylated in the two culture conditions (Fig. 1D). Flow cytometry analysis showed that over 85% of cells were OCT3/4 positive under both iMatrix511 + StemFit and LN521 + mTeSR culture conditions (Fig. 1E). The expression of mRNA differentiation marker is affected by different iPSC culture media using TaqMan Array Human Stem Cell Pluripotency. Pluripotency reveals that iPSCs cultured under LN521 + mTeSR conditions expressed higher mRNA levels of endoderm marker SOX17 and mesoderm marker Brachyury (T) than iMatrix511 + StemFit conditions (Fig. 1F). On the other hand, iPSCs cultured under LN521 + mTeSR conditions expressed lower mRNA levels of ectoderm marker PAX6 than iMatrix511 + StemFit conditions (Fig. 1F). These results showed that changing the combination of medium and scaffold material maintained the pluripotency of iPSCs but altered the tendency of the cells to differentiate.

### The components of the pre-culture medium influence the differentiation of three germ layers

**Fig. 2:**
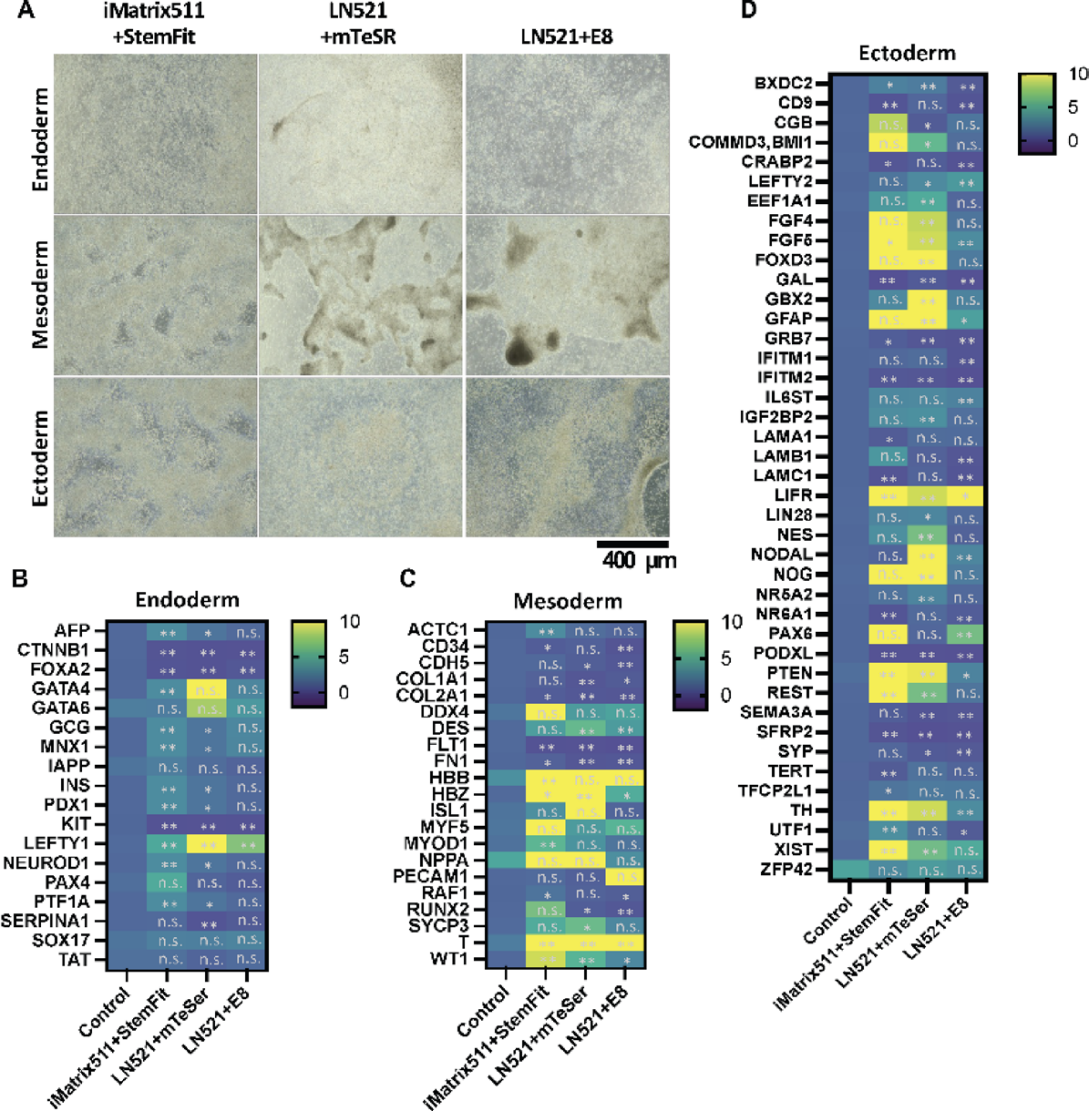
The effects of pre-culture were compared in the induction of trilineage differentiation. Three different methods, iMatrix511 + StemFit, LN521 + mTeSR, and Essential 8, were used to pre-culture the iPSCs. (**A**) iPSCs were induced to differentiate into three germ layers for 2 days using the STEMdiff Trilineage Differentiation Kit (STEMCELL Technologies). The panels show images of optical micrographs. Scale bar = 400 μm. The iPSCs were seeded at a concentration of 5,000 cells per well into six wells. The culture conditions were iMatrix511 + StemFit, LN521 + mTeSR, and LN521 + E8. On day seven after seeding the cells, differentiation into the three germ layers (ectoderm, mesoderm, and endoderm) was induced using the STEMdiff Trilineage Differentiation Kit (STEMCELL Technologies). For the control group, iPSCs that had not been induced to differentiate using iMatrix511 + StemFit were used. On the second day after differentiation induction, mRNA was extracted, and cDNA was synthesized. The results of the mRNA expression analysis are shown. Expression was calculated using the ΔΔCt method. Target gene expression was corrected based on the expression of housekeeping genes. The average mRNA expression in the controls was normalized to 1. The real-time quantitative PCR (qPCR) results for the endoderm (**B**), mesoderm (**C**), and ectoderm (**D**) are shown (*n* = 6).

The effect of pre-culture was compared with that of trilineag differentiation induced in 2 days using the STEMdiff Trilineage Differentiation Kit (STEMCELL Technologies) (Fig. 2A). For the control group, iPSCs that had not been induced to differentiate using iMatrix511 + StemFit were used. On the second day after differentiation induction, mRNA was extracted, and cDNA was synthesized. The results of the mRNA expression analysis are shown. The average mRNA expression in the controls was normalized to 1. The real-time quantitative PCR (qPCR) results for the endoderm (Fig. 2B), mesoderm (Fig. 2C), and ectoderm (Fig. 2D) are shown. Left-right determination factor 1 (LEFTY1), the gene’s role in determining the left-right asymmetry of the heart is significant^51^in the endoderm was higher when LN521 + mTeSR was used in the pre-culture (Fig. 2B). The mRNA expression level of Desmin (DES) mRNA, a type III intermediate filament that is specific to muscle, including cardiac muscle^52^was high (Fig. 2C). PAX6 mRNA expression, an endoderm marker, which is inaccurate. PAX6 is a well-established marker of ectodermal, especially neuroectodermal lineage commitment. PAX6 was higher when iMatrix511 + StemFit and LN521 + E8 was used in preculture (Fig. 2D). These results indicate that the results of early differentiation induction of iPSCs are influenced by the characteristics of the culture environment (medium and scaffold material) prior to the use of the differentiation induction medium.

### Pre-culture medium influences subsequent myocardial tissue differentiation

**Fig. 3:**
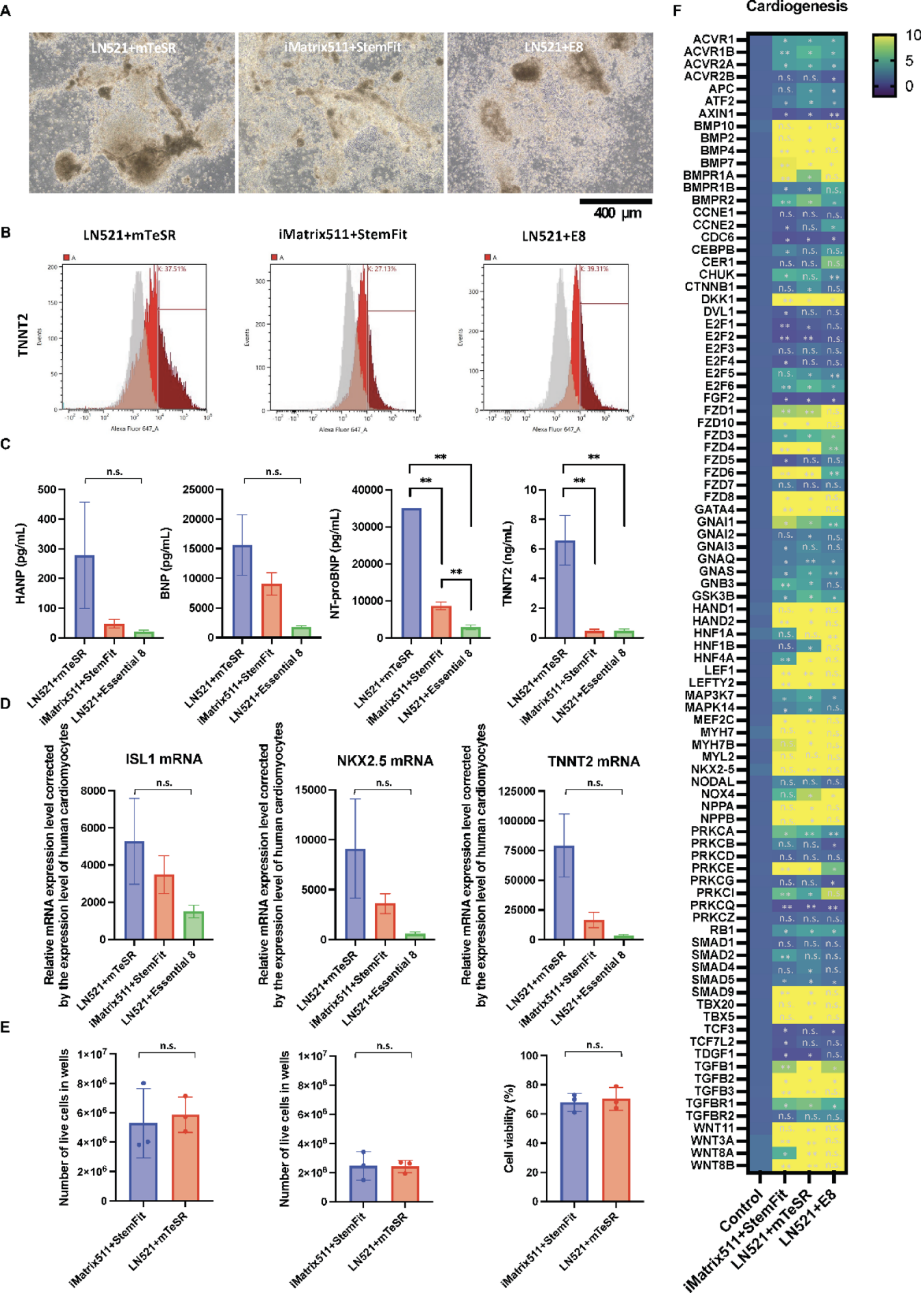
The potential to form cardiomyocytes was evaluated. Three different methods, iMatrix511 + StemFit, LN521 + mTeSR, and Essential 8, were used to pre-culture the iPSCs. Cardiomyocyte differentiation was induced using the PSC Cardiomyocyte Differentiation Kit (Thermo Fisher Scientific). (**A**) The panels show images of optical micrographs. Scale bar = 400 μm. (**B**) Results of flow cytometry analysis of the TNNT2-positive rate of iCM. The TNNT2 positive rate of iCM cultured with iMatrix511 + StemFit was 37.5%, LN521 + mTeSR was 27.1%, and Essential 8 were used in the pre-culture after LN521 + mTeSR was 39.3%. The corresponding isotype is shown in gray. The protein expression level of the cardiac marker TNNT2 was increased by approximately 2% when Essential 8 was used in the pre-culture after LN521 + mTeSR. (**C**) When the culture supernatant was concentrated 10-fold with Amicon-Ultra15 (10 K) (Merck Millipore) and subjected to heart-related assays, the levels of cardiac secreted hormones such as ANP, BNP, NT-proBNP and TNNT2 were also increased in iCM cultured with LN521 + mTeSR. *n* = 3. Data represent the mean S.D. **P < 0.01. (**D**) The mRNA levels of cardiac progenitor cell markers *ISL1* and *NKX2.5* were higher when LN521 + mTeSR was used. *n* = 6. Data represent the mean S.D. (**E**) Total number of cells (left panel), number of dead cells (middle panel), number of live cells (right panel) in the iCM cultured with iMatrix511 + StemFit or LN521 + mTeSR. *n* = 3. Data represent the mean S.D. (**F**) mRNA expression analysis using TaqMan Array Human Factors for Cardiogenesis. The earliest expressed transcription factors that initiate cardiac fate are the homeobox transcription factor NKX2.5 (NK2 Transcription Factor Related Locus-5) and members of the GATA (GATA Binding Protein) family of zinc finger transcription factors including, GATA4 (GATA Binding Protein-4). The mRNA expression increased more than 10-fold compared to the control (no differentiation induction) is shown in yellow. All cardiomyocytes induced to differentiate from iPSCs cultured in three different pre-culture media showed a more than 10-fold increase in both NKX2.5 and GATA4 mRNA expression levels compared to controls prior to the start of differentiation induction. *n* = 3.

An examination was conducted on the formation of cardiac tissue under adhesive culture conditions. Cardiac differentiation was induced using the PSC Cardiomyocyte Differentiation Kit (Thermo Fisher Scientific) (Fig. 3A). Light microscopy revealed clear adherent cells and a brown, raised histomorphology rich in fibrous components in all culture conditions (Fig. 3A). Troponin T positivity was determined by flow cytometric analysis. Troponin T positivity was 37.5% for LN521 + mTeSR, 27.1% for iMatrix511 + StemFit and 39.3% for LN521 + E8 (Fig. 3B). Protein expression of the cardiac marker TNNT2 was increased by approximately 2% when E8 was used in the pre-culture. When the culture supernatant was concentrated 10-fold using Amicon-Ultra15 (10 K) (Merck Millipore) and subjected to cardiac-related assays, higher levels of cardiac hormones such as ANP and BNP were detected in LN521 + mTeSR than in other culture conditions, and significantly higher levels of NT-proBNP and TNNT2 were detected (Fig. 3C). These results indicate that cardiac tissue formation is occurring. When LN521 + mTeSR was used in pre-culture, mRNA for the cardiac progenitor markers ISL1 and NKX2.5 as well as the cardiac marker TNNT2 was higher (Fig. 3D). There was no effect on the total number of cells, dead cells, or live cells in iCMs cultured in iMatrix511 + StemFit or LN521 + mTeSR (Fig. 3E). The results of mRNA expression analysis using a PCR panel for factors related to heart formation are shown in the heat map (Fig. 3F).

### TNNT2 positivity in subsequent myocardial differentiation is increased using E8 in preculture

**Fig. 4:**
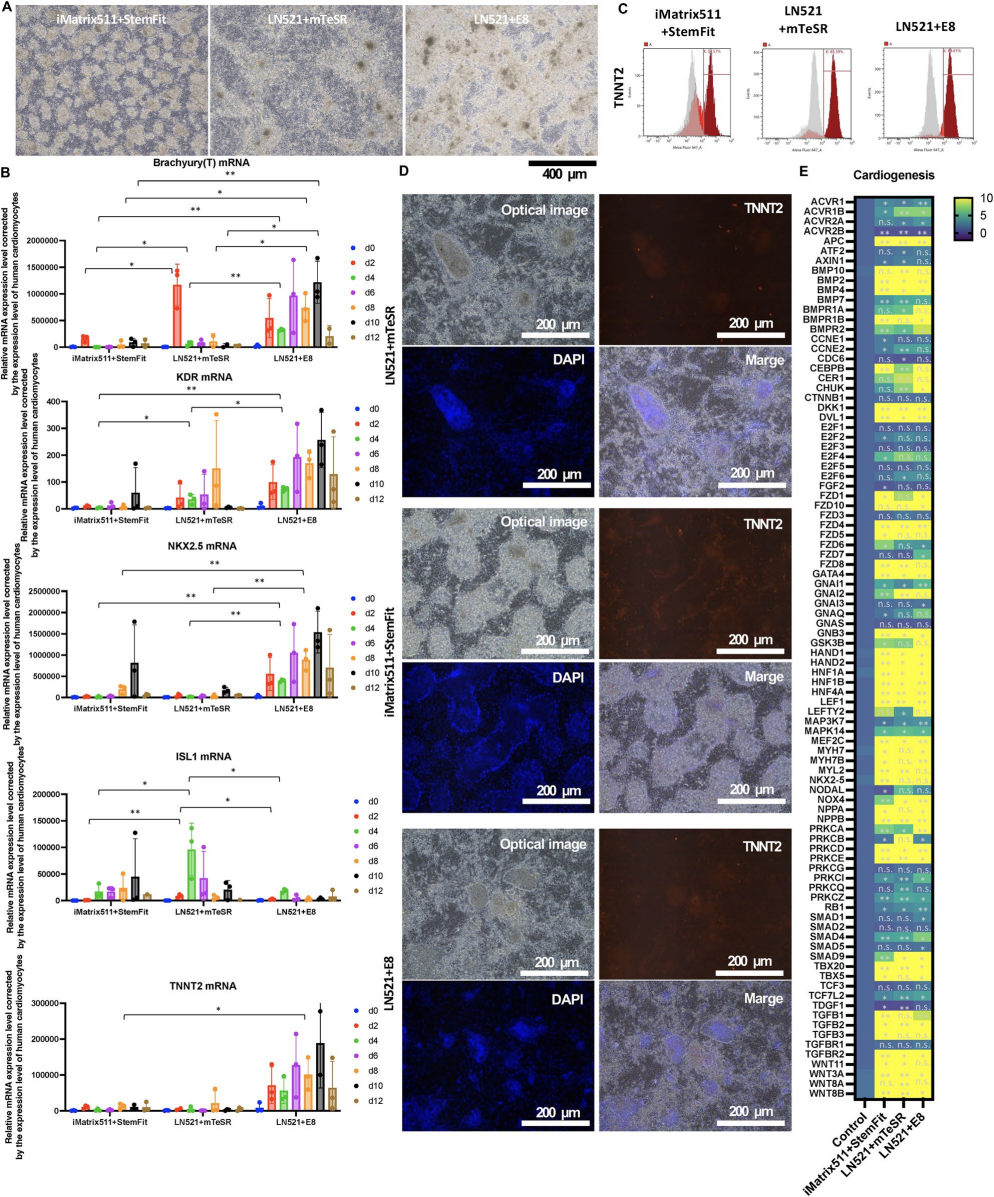
The potential to form heart tissue was evaluated. Three different methods, iMatrix511 + StemFit, LN521 + mTeSR, and Essential 8, were used to pre-culture the iPSCs. Cardiomyocyte differentiation was induced using the STEMdiff Cardiomyocyte Differentiation and Maintenance Kits (STEMCELL Technologies). (**A**) The panels show images of optical micrographs. Scale bar = 400 μm. (**B**) The mRNA expression levels of T (Brachyury), KDR, NKX2.5, ISL1, TNNT2 (cTnT) at 0, 2, 4, 6, 8, 10, and 12 d after the start of differentiation induction are shown. Expression was calculated using the ΔΔCt method. The expression of the target gene was corrected for the expression of the housekeeping gene. Relative values are shown. *n* = 3. Data represent the mean S.D. (**C**) Results of flow cytometry analysis of the TNNT2-positive rate of iCM at 12 days after the start of differentiation induction. The TNNT2 positive rate of iCM cultured with iMatrix511 + StemFit was 54.6%, LN521 + mTeSR was 85.4% and Essential 8 were used in the pre-culture after LN521 + mTeSR was 88.6%. The corresponding isotype is shown in gray. The protein expression level of the cardiac marker TNNT2 was increased when Essential 8 was used in pre-culture after LN521 + mTeSR. (**D**) Three different methods, iMatrix511 + StemFit, LN521 + mTeSR, and Essential 8, were used to pre-culture the iPSCs. Cardiomyocyte differentiation was induced using the STEMdiff Cardiomyocyte Differentiation and Maintenance Kits (STEMCELL Technologies). iCM were subjected to TNNT2 and Hoechst staining and fluorescence microscopy on day 12 after differentiation induction. Scale bar = 200 μm. (**E**) mRNA expression analysis using TaqMan Array Human Factors for Cardiogenesis. The mRNA expression increased more than 10-fold compared to the control (no differentiation induction) is shown in yellow. All cardiomyocytes induced to differentiate from iPSCs cultured in three different pre-culture media showed a more than 10-fold increase in both NKX2.5 and GATA4 mRNA expression levels compared to controls prior to the start of differentiation induction. *n* = 3.

Myocardial differentiation was assessed using the STEMdiff Ventricular Cardiomyocyte Differentiation Kit (STEMCELL Technologies). Prominent brown cells were observed in all culture conditions (Fig. 4A). Flow cytometric analysis showed that TNNT2 positivity was 54.6% for iMatrix511 + StemFit, 85.4% for LN521 + mTeSR, and 88.6% for LN521 + E8 (Fig. 4C). Fluorescence immunostaining confirmed that these ridge cells were TNNT2 positive (Fig. 4D), ANP positive (Sup. Figure 2 A), and proBNP positive (Sup. Figure 2B). The mRNA expression of the process of induction of differentiation into cardiomyocytes was examined at days 0, 2, 4, 6, 10, and 12. mRNA expression of the mesoderm marker Brachyury(T) mRNA was transiently expressed in LN521 + mTeSR and iMatrix511 + StemFit at day 2, but was persistently expressed in LN521 + E8, showing a sustained increase in expression from day 2-10 (Fig. 4B). TNNT2 mRNA, a marker of cardiomyocytes, showed a sustained increase in expression from day 2-10 in LN521 + E8 (Fig. 4B). These results indicated that LN521 + E8 repeatedly induced sustained myocardial differentiation from day 2-10. The results of mRNA expression analysis using a PCR panel for factors related to heart formation are shown in the heat map (Fig. 4E).

### Clinical transplant preculture media also affect mRNA expression of differentiation markers

StemFit AK03N (Ajinomoto) was selected as the material to be used for clinical cell production, and seven types of preculture media were prepared by changing the formulation of its liquid A, B, and C. StemFit AK03N (Ajinomoto) is a growth medium for embryonic stem (ES) and iPSCs that was developed jointly by Ajinomoto and the center for iPS Cell research and application (CiRA) at Kyoto University, the predecessor of our organization. The component-adjusted StemFit AK03N medium is commonly used in academic research at CiRA and our institution for manufacturing cells for clinical use from iPSCs. For example, the StemFit AK03N medium without C medium is often used as a base for blood cell cultures. The StemFit AK03N C medium contains basic fibroblast growth factor (bFGF)^53^. The StemFit AK03N medium without C medium has long been used to culture somatic cells prior to reprogramming^54^ as well as for in vitro differentiation^38^. Thus, the StemFit AK03N medium allows for the creation of different media formulations by combining liquids A, B, and C. In this study, we demonstrated that differences in StemFit, mTeSR, and E8 media affect the efficiency of inducing cardiac tissue differentiation. However, we considered the possibility that other factors, such as the combination of medium and scaffold materials and the concentrations of various medium components and additives, could impact the results. Therefore, we set up conditions in which only the composition of StemFit AK03N (Ajinomoto), Liquid A, B, and C changed. We then investigated the effects of the culture media on the efficiency of inducing differentiation into cardiac tissue.

**Fig. 5:**
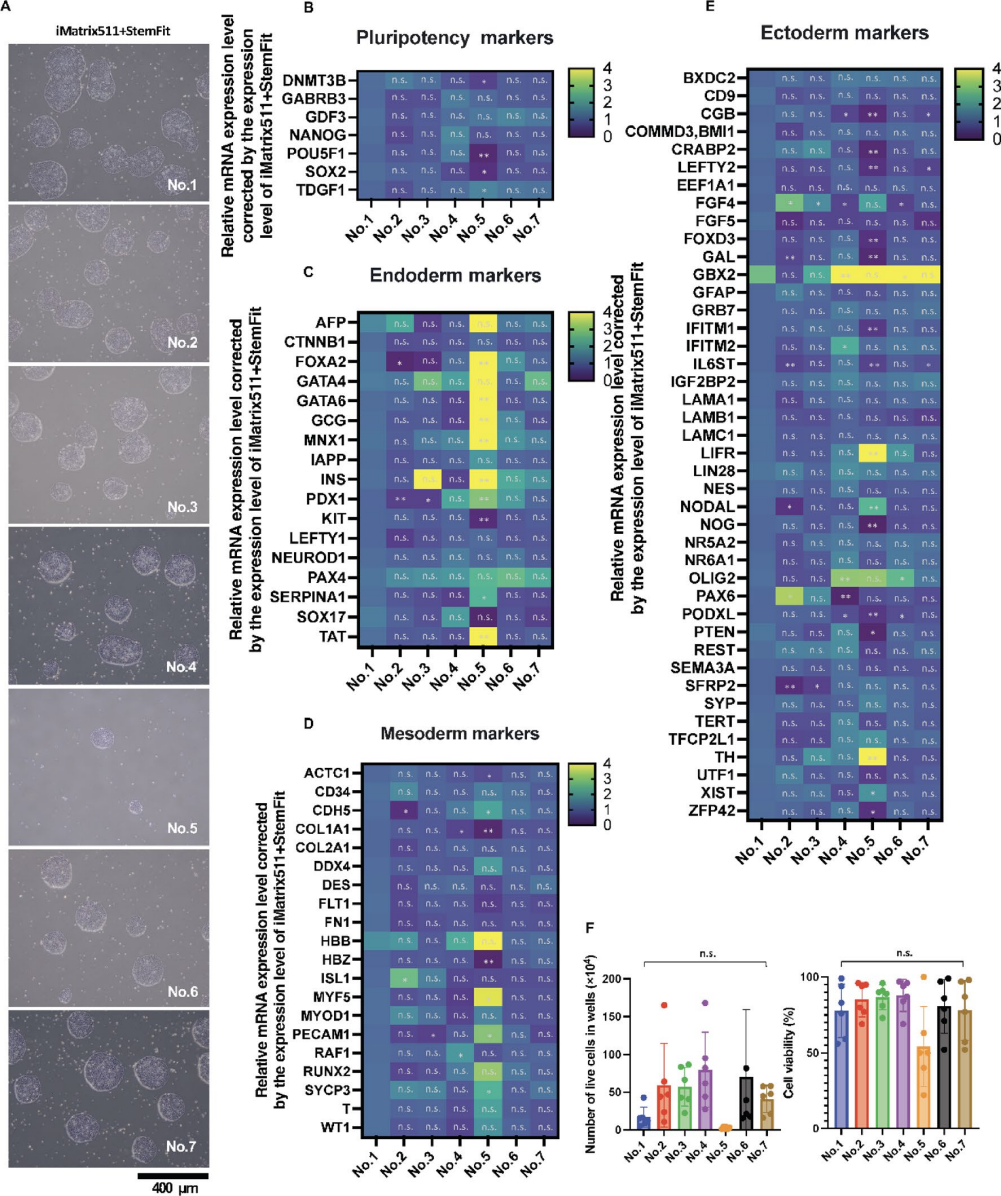
Quality assessment of iPSCs cultured with clinical grade preculture media. Clinical grade iPSC culture media with seven different mixing ratios (StemFitAK03:No.1-7) were prepared from StemFitAK03N. iPSCs cultured with StemFitAK03:No.1-7 medium. (**A**) Optical microscopy images taken at day 6 a after seeding at 1.3 × 10^4^ cells/well (6 well). Scale bar = 400 μm. (**B**) StemFit AK03: No. 1, a commercial composition, was used as a control. The average mRNA expression in the controls was normalized to 1. The real-time quantitative PCR (qPCR) results for the endoderm (**C**), mesoderm (**D**), and ectoderm (**E**) are shown. *n* = 4. (**F**) The number of live cells in the iPSC at day 6 a after seeding at 1.3 × 10^4^ cells/well (6 well) (left panel) and cell viability (right panel). *n* = 6. Data represent the mean S.D.

StemFit AK03: No. 1 was prepared at commercial ratios, StemFit AK03: No. 2-3 at low nutrient levels such as Essential 8, and StemFit AK03: No. 4-7 at high nutrient levels such as mTeSR. The quality of the iPSCs was compared to clinical preculture media. Colony morphology was ES cell-like in all preculture media StemFit AK03: No.1-7 (Fig. 5A). Expression of mRNA of undifferentiated markers. StemFit AK03: No. 1, a commercial composition, was used as a control. POU5F1 and SOX2 mRNA expression was lower in StemFit AK03: No. 5 than in StemFit AK03: No. 1 (Fig. 5B). StemFit AK03: No. 5 showed increased mRNA expression for endoderm (Fig. 5C), mesoderm (Fig. 5D), and ectoderm (Fig. 5E), and cell proliferation was slow, and the number of colonies was low (Fig. 5A). The mRNA expression level of ISL3, a marker of cardiac progenitor cells and an indicator of differentiation into the mesodermal lineage differentiation, was significantly higher in StemFit AK03: No. 2 than in StemFit AK03: No. 1(Fig. 5D). StemFit AK03: No. 5 had lower cell numbers and cell viability than the other six media types (Fig. 5F).

### Increased TNNT2 positivity was observed in both StemFit AK03: No.2 and No.3 of the Essential 8-like composition of the clinical pre-culture medium

**Fig. 6:**
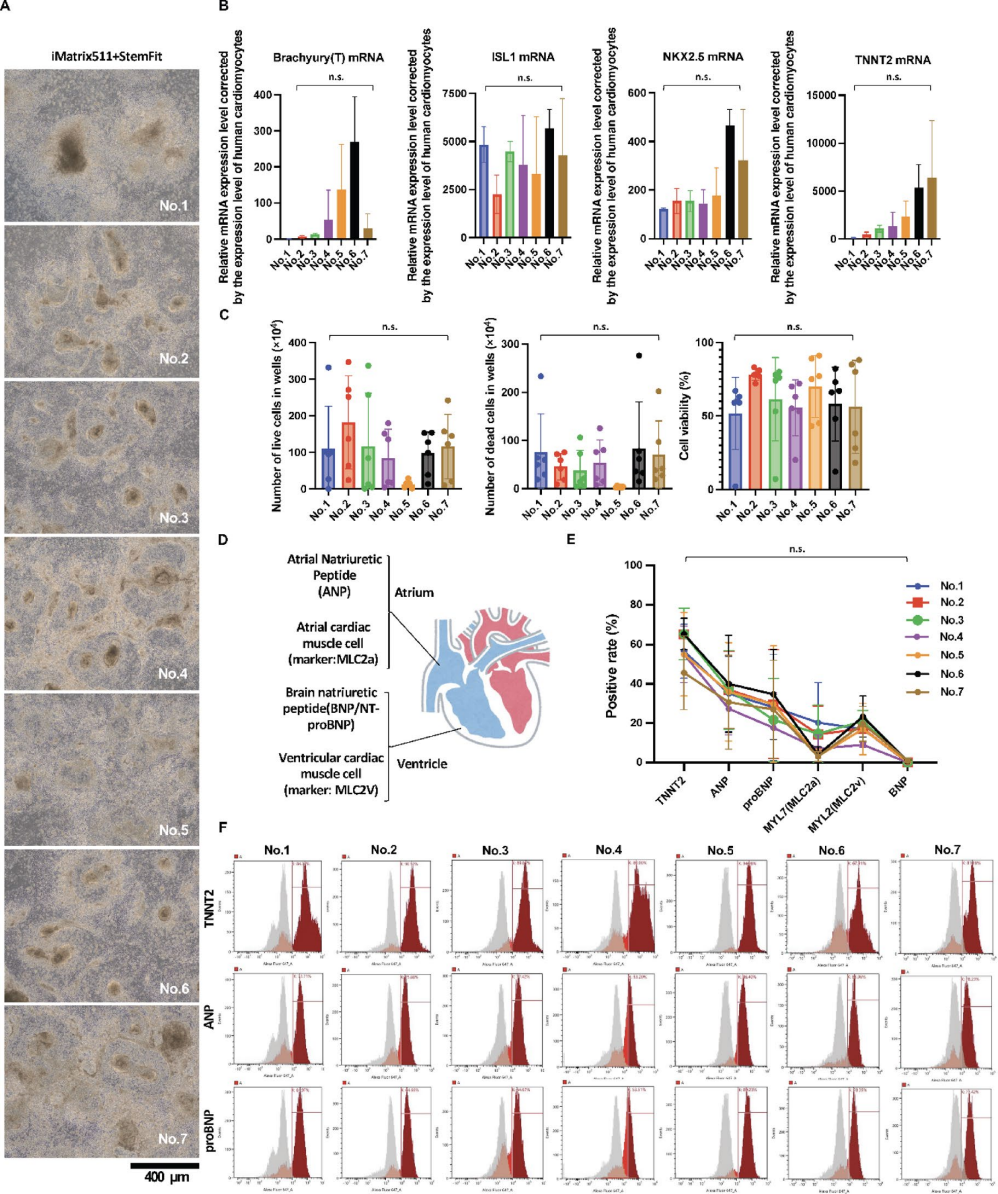
The potential to form heart tissue was evaluated by using PSCs cultured with clinical grade preculture medium. Seven different methods, iMatrix511 + StemFitAK03:No.1-7, were used to culture the iPSCs. Cardiomyocyte differentiation was induced using the customized media. (**A**) The panels show images of optical micrographs. Scale bar = 400 μm. (**B**) Shown are the mRNA levels of the mesoderm marker *Brachyury(T)*, the cardiac progenitor markers *ISL1* and *NKX2.5*, and the cardiac marker *TNNT2* at day 12 of differentiation induction. *n* = 3. Data represent the mean S.D. (**C**) The number of live cells (left panel), the number of dead cells (middle panel), the cell viability (right panel) in the iCM. *n* = 6. Data represent the mean S.D. (**D**) The illustration shows various site-specific markers of the heart. (**E**) Seven different methods, iMatrix511 + StemFitAK03:No.1-7 were used to culture the iPSCs. Cardiomyocyte differentiation was induced with the custom-made media. Results of flow cytometric analysis of the TNNT2, ANP, proBNP, MYL7(MLC2a), MYL2(MLC2v) and BNP positive rate of iCM. The TNNT2 positive rate of iCM cultured with StemFitAK03: No. 1 (commercial StemFit AK03 medium) had a troponin positivity of 84%, StemFitAK03: No. 3 had a positivity of 89%, StemFitAK03: No. 2 had a positivity of 91%, and StemFitAK03: No. 5 increased to 95%. *n* = 3 technical replicates. Error bars, S.E. It indicated the representative value of the technical replicates. (**F**) Representative histograms of each flow cytometry result are displayed.

Cardiomyocyte differentiation was evaluated in the clinical pre-culture medium. The following custom differentiation induction media were used to induce cardiomyocyte differentiation. Cardiomyocyte Differentiation Induction Medium (collected on day 12) Day 0 12 µM CHIR99021, RPMI/B-27 without insulin; Day 1 RPMI/B-27 without insulin; Day 3 2 µM XAV939, RPMI/B-27 without insulin; Day 5 RPMI/B-27 without insulin; Day 7 RPMI/B-27 with insulin. Photographs of cardiomyocytes induced to differentiate after culture in clinical preculture medium No. 1-7 on day 12 (Fig. 6A). Number of viable cells (Fig. 6C, left panel), number of dead cells (Fig. 6C, middle panel) and cell viability (Fig. 6C, right panel) on day 12. Figure showing the tissue expression sites of the markers in cardiac tissue (Fig. 6D). The percentages of cells for various markers measured by flow cytometry are tabulated and graphically depicted (Fig. 6E). There were no statistically significant differences in the expression levels of various markers in clinical preculture medium No. 1-7. However, the occurrence of TNNT2-negative waveforms was suppressed in Nos. 2, 3, and 5 compared to No. 1 (the commercial medium) in the waveform of the TNNT2 histogram. Representative histograms of each flow cytometry result are displayed (Fig. 6F). No. 6, which showed an increase in histogram waveforms in TNNT2-negative areas from No.1 (Fig. 6F), showed a trend toward higher expression of the mesoderm marker Brachyury(T), the cardiac progenitor marker NKX2.5, and the cardiac marker TNNT2 in mRNA expression at day 12 of differentiation induction (Fig. 6B). This suggests a delayed expression of mRNAs expressed by young cells even during the maturation phase of differentiation induction. In StemFit AK03: No.2 and No.3, which showed a decrease in histogram waveforms in TNNT2-negative areas from No.1 was detected by flow cytometry (Fig. 6F), mRNA expression on day 0 of differentiation induction showed a trend toward higher expression of the cardiac progenitor marker ISL1 (Fig. 5D). These results suggest that increased expression of ISL1 increased the percentage of TNNT2-positive cells.

### Expression of cardiac tissue markers is decreased with organization inhibition

In previous chapters, we evaluated the effect of pre-culture media on the induction of cardiac tissue differentiation using media suitable for clinical cell production. Next, we examined the characteristics of cells that easily detach from the culture plate and how to properly handle them during the monophasic induction process used in this study to induce cardiac differentiation. Customized differentiation induction media were used to induce cardiomyocyte differentiation. Biolaminin 521 LN was used as the scaffold material, and when LN521 + mTeSR (control) was used to induce the differentiation into cardiomyocytes, a layer of adherent cells and clusters of elevated cells was observed (Sup. Figure 1 A, upper left panel). Sup. Figure 1 A (lower left) shows non-adherent cells. After removing as many non-adherent cells as possible, the adherent cells displayed a leaf-like structure, and a brownish cell mass appeared in the center (Sup. Figure 1 A, upper right). When the cell layer was dissociated into single cells by trypsinization (trypsinized cells), the dissociated cells could not repair themselves and the leaf-like structure disappeared (Sup. Figure 1 A, bottom right panel). Notably, the positivity of all markers was lower only in adherent cells only (Sup. Figure 1 A, upper right panel), in which nonadherent cells were removed, then in the control (Sup. Figure 1B), especially MLC2a-positive cells were no longer observed (Sup. Figure 1B). This result indicates that non-adherent cells contain average 17.5% ANP-positive cells. This suggests that atria-forming cells may be abundant in non-adherent cells during the period of cardiac tissue formation.

## Discussion

Various methods to induce cardiomyocyte differentiation from pluripotent stem cells such as iPSCs have been reported and reviewed by Lyra-Leite et al.^55^. However, there are few reports on the importance of ANP and BNP expression during the formation of cardiomyocytes artificially induced to differentiate from iPSCs, and no references were obtained. In this study, we demonstrated that ANP-positive cells become floating cells during the cardiac differentiation process and promote the formation of cardiac tissue (see Sup. Figure 1 A and Sup. Figure 1B). By changing the composition of the iPSC preculture medium to that of Essential 8-like medium, it was possible to enhance tissue induction in the heart (Fig. 6F). A possible reason for this is that the Essential 8-like medium produced more raised brown ANP-positive cells (Fig. 2A). An interesting report on this phenomenon is the report by Shiraki et al. showed^56^ that lowering the NANOG mRNA expression level of pluripotent stem cells leads to a 3-fold increase in their subsequent ability to differentiate into three germ layers. In fact, NANOG mRNA expression levels were significantly lower in iPSCs cultured in StemFitAk03: No. 2 (Essential 8-like medium) than in StemFitAk03: No. 4 (which had the lowest ANP protein expression levels after cardiomyocyte differentiation (Fig. 6E)) (Fig. 5B (numbers not shown)). The interpretation that low expression of NANOG in pre-culture promotes cardiac organization during cardiomyocyte differentiation and increases the percentage of ANP-positive cells is consistent with experimental results using the iMatrix-511 scaffold material (Figs. 5 and 6).

On the other hand, Yu et al. reported that FGF2 signaling maintains NANOG expression through the MEK-ERK pathway and induces the expression of the mesoderm marker T (brachyury) in ES cells^57^. Next, Asai et al. reported that at the lateral plate mesoderm stage BMP signaling induces cardiomyocyte differentiation, while FGF inhibits the induction of cardiomyocyte differentiation and promotes differentiation into vascular endothelial cells^58^. From these reports, it is concluded that FGF2 added to the iPSC culture medium enhances the induction of cardiomyocyte differentiation when it is rapidly reduced after the expression of the mesoderm marker T (Brachyury), and that high concentrations of FGF2 induce differentiation into vascular endothelial cells. The induction of cardiomyocyte differentiation is enhanced when the expression of FGF2 is reduced immediately after the expression of Brachyury. Looking at the experiment in Fig. 4B, which analyzed the time course of the differentiation induction process of cardiomyocytes, a transient increase in T (Brachyury) mRNA expression was observed in LN521 + mTeSR and iMatrix511 + StemFit on day 2, the start of differentiation induction. To interpret these results, we focused on the medium composition of the iPSCs. mTeSR and StemFit contain recombinant human albumin^59^which is a nutrient for iPSCs to secrete heparan sulfate proteoglycans (HSPG), collagen IV and collagen I. HSPG and collagen I stabilize FGF2^60^ and increase the activity of FGF2 and FGF receptors. On the other hand, Essential 8 does not contain recombinant human albumin and which requires a higher concentration of FGF2, as this molecule alone maintains the activity of the FGF receptor. As a result, the use of Essential 8-like medium as a preculture medium would reduce the effect of FGF2 quickly after the start of differentiation induction.

The cardiomyocyte differentiation induction protocol used in this study primarily involves a medium composition suitable for cardiac ventricular differentiation^55^. Inhibition of FGF signaling has been reported to result in ectopic atrial and ventricular gene expression in ventricular myocytes^61^. We investigated the protein expression of an atrial marker (MLC2a) and a ventricular marker (MLC2v). iPSCs cultured in preculture medium were induced to differentiate into cardiomyocytes; StemFitAk03: No.2-3 (Essential 8-like) had high MLC2a expression levels (average 14.5%), while StemFitAk03: No.4-7 (mTeSR-like) had low MLC2a expression levels (average 4.3%) (Fig. 6E). Burridge et al. reported that the expression of the myocardial marker TNNT2 and the atrial marker MLC2a reaches > 80% on day 10 of differentiation induction. Meanwhile, the expression of the ventricular marker MLC2v is reported to be less than 10% on day 10 and reaches 60% between days 30 and 60^43^. Therefore, the protein expression levels of the ventricular marker MLC2v, as shown in the data of this study, should be interpreted as provisional values obtained early after differentiation induction. The results showed that the composition of the pre-culture medium even affected the pattern of formation of the atria and ventricles of the heart.

In conclusion, in this study, the composition of the preculture medium was varied when inducing differentiation of iPSCs: the Essential 8-like preculture medium promoted the formation of TNNT2+, ANP+, and proBNP + ridge cells and increased ANP-expressing cells, and it also promoted cardiac tissue formation, which increased the percentage of TNNT2-positive cells.

## Supplementary Information

Below is the link to the electronic supplementary material.

Supplementary Material 1

Supplementary Material 2

## Data Availability

The datasets generated and/or analysed during the current study are available in the Gene Expression Omnibus (GEO) repository, GSE296185 (https://www.ncbi.nlm.nih.gov/geo/query/acc.cgi?acc=GSE296185). Raw data were generated at CiRA Foundation. Derived data supporting the findings of this study are available from the corresponding author Yoshiki Nakashima (yoshiki.nakashima@cira-foundation.or.jp) on request.
