# Supplementary figures and images for: Effect of iPS cell culture medium on the differentiation potential of induced cardiac tissues
Source: Sci Rep. 2025 Aug 3;15:28301. doi: 10.1038/s41598-025-13259-x (PMC12319073; doi:10.1038/s41598-025-13259-x)

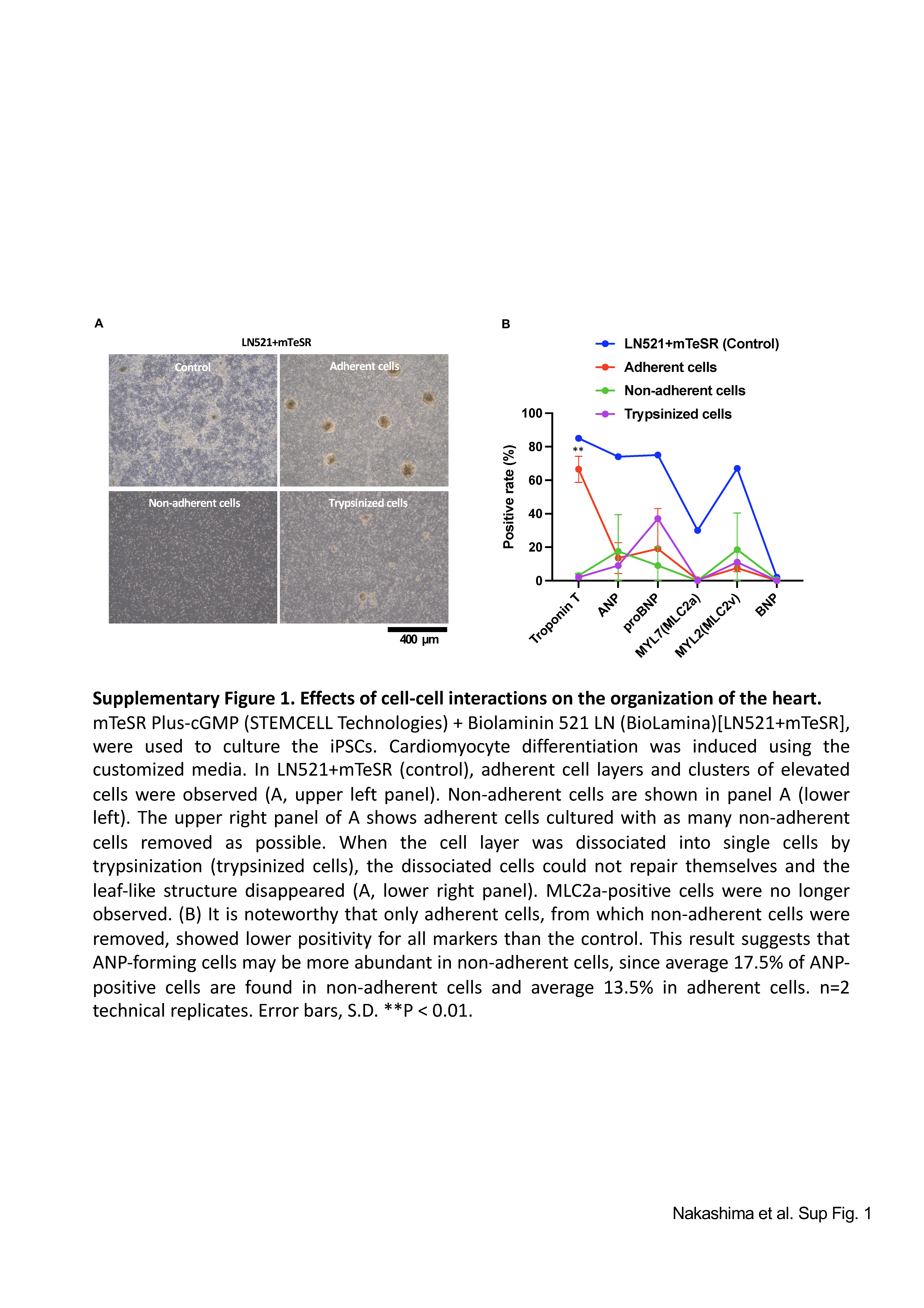

Supplement: Supplementary file 1 — Supplementary Material 1 [file 41598_2025_13259_MOESM1_ESM.tiff]

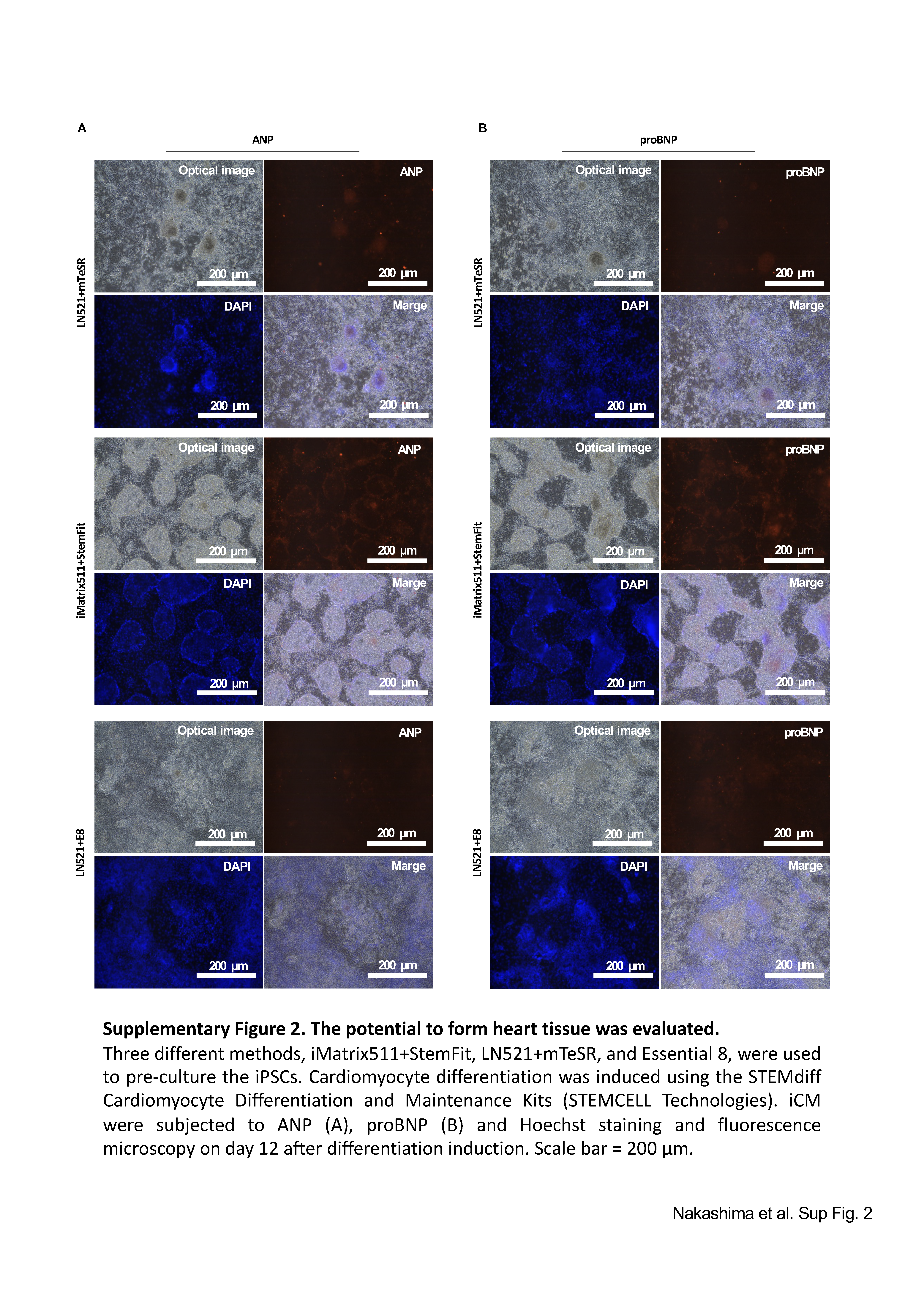

Supplement: Supplementary file 2 — Supplementary Material 2 [file 41598_2025_13259_MOESM2_ESM.tiff]
